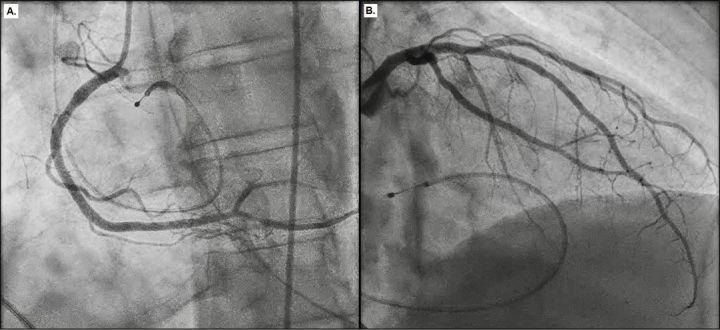# A case of Epstein-Barr virus–associated pericarditis progressing to complete atrioventricular block and cardiac arrest

**DOI:** 10.1016/j.hrcr.2023.07.010

**Published:** 2023-07-23

**Authors:** Hafez Golzarian, David Eapen, Fnu Sandesh, Shudipan Chakraborty, Megan Hennon, Jannat Bux, Jason Kim, Aakash Kumar, Sandeep M. Patel

**Affiliations:** ∗ Internal Medicine Residency Program, Mercy Health—St. Rita’s Medical Center, Lima, Ohio; † Department of Internal Medicine, Jinnah Sindh Medical University, Karachi, Pakistan; ‡ Internal Medicine Residency Program, Mercy Health—St. Vincent’s Medical Center, Toledo, Ohio; § Kentucky College of Osteopathic Medicine, Pikeville, Kentucky; ¶ Structural Heart and Interventional Cardiology—St. Rita’s Medical Center, Lima, Ohio

**Keywords:** Heart block, Cardiac arrest, Epstein-Barr, STEMI, Myocardial infarction, Arrhythmia, Angina

**Key Teaching Points**

• High-degree atrioventricular block with progression to complete heart block and cardiac arrest is a potential cardiac manifestation of subacute Epstein-Barr virus infection.
• Coronary vasospasm as a concomitant manifestation of cardiac inflammation seen in conditions such as myocarditis and even pericarditis can lead to sudden death.
• Definitive management of such patients with refractory viral pericarditis with such electrical conduction disturbances involves permanent pacemaker implantation.

## Introduction

Epstein-Barr virus (EBV) is a very rare cause of pericarditis, with only a handful of cases reported in the literature.^1^, ^2^, ^3^, ^4^ Despite an overall mortality rate of only 1.1%, pericarditis can progress to life-threatening complications such as cardiac tamponade, purulent pericarditis, or pericardial effusion.^5^ Owing to lack of literature and large-scale studies, these rare life-threatening cardiac manifestations of EBV are poorly understood in the clinical setting. We present the case of an immunocompetent male who presented with infectious mononucleosis due to EBV. Three months later, he presented again with pericarditis and respiratory failure, which rapidly progressed to cardiac arrest. Upon successful resuscitation, thorough review of his telemetry revealed that he developed high-degree atrioventricular (AV) heart block, which rapidly progressed to complete heart block with bradycardia followed by asystole. To our knowledge this is the first reported case of EBV pericarditis progressing to third-degree heart block and cardiac arrest. Our case elucidates this underrecognized life-threatening manifestation of EBV-induced pericarditis and urges clinicians to recognize the potential for such patients to rapidly decompensate.

## Case report

**Figure 1:**
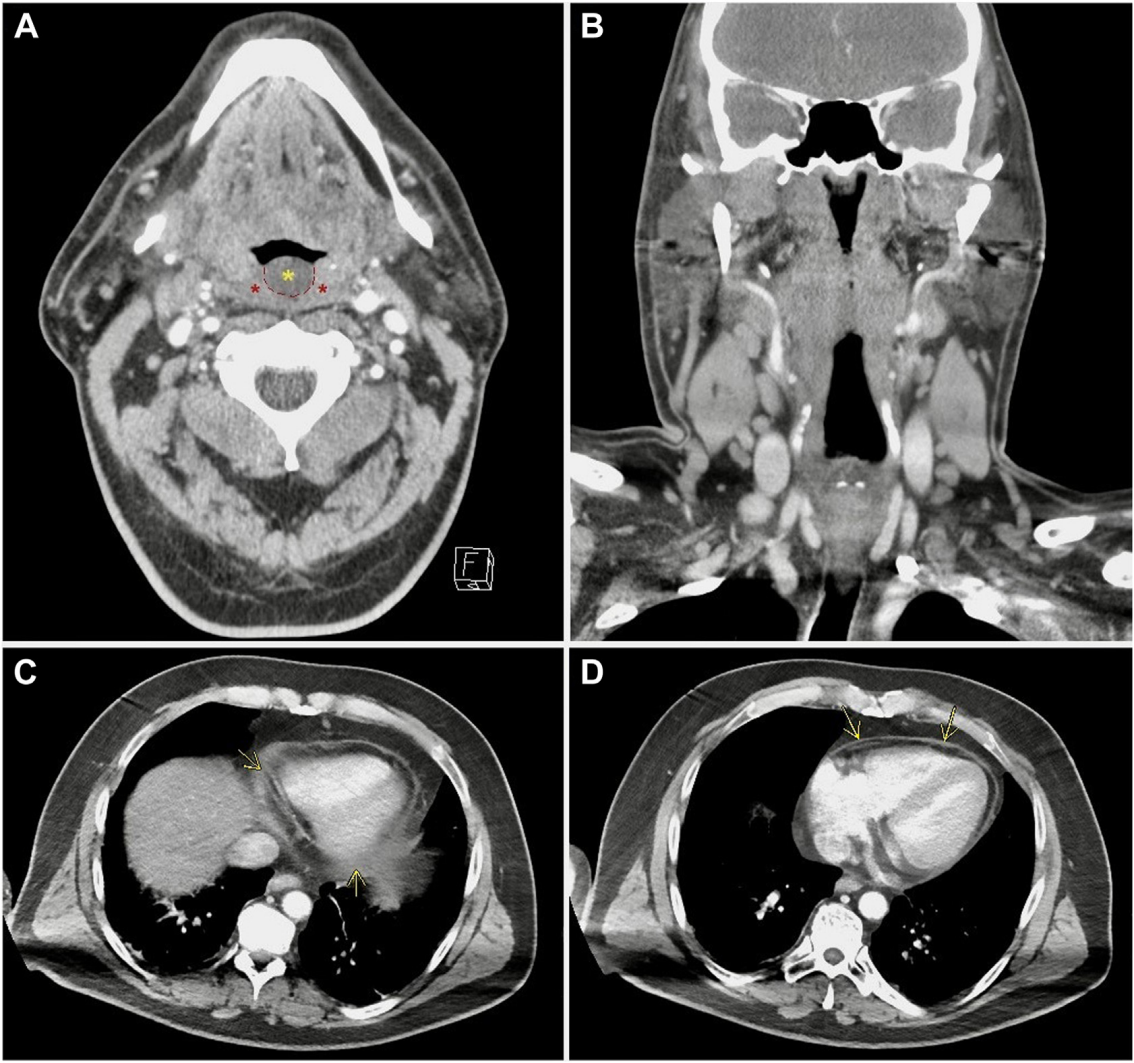
**A:** Computed tomography with intravenous contrast with a transverse view revealing bilateral thickened and edematous tonsils (red asterisks) and uvula (yellow asterisk). **B:** Coronal view revealing concomitant pronounced reactive cervical lymphadenopathy expected in Epstein-Barr virus infection. **C, D:** Transverse views of the mediastinum revealing new trace pericardial effusion with enhancement and thickening of the pericardial sac (*yellow arrows*).

**Supplemental Figure 1:**
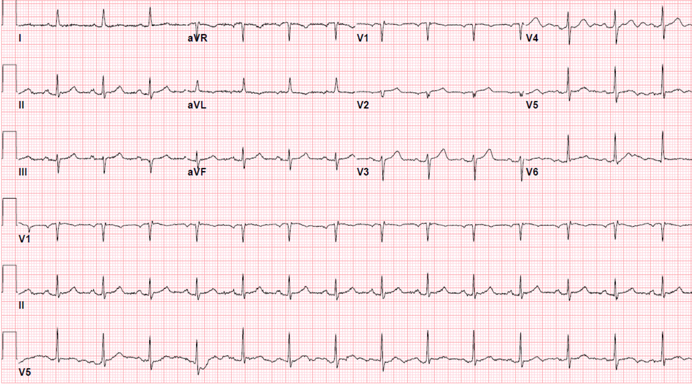


A 52-year-old gentleman with a medical history of diabetes mellitus presented to the emergency department with new-onset exudative pharyngitis and fatigue. Workup was inconclusive, and he was provided with supportive care and discharged home with amoxicillin-clavulanate. Three months later he presented again with sudden-onset sharp chest pain that radiated to his neck and shoulders bilaterally. Chest pain was relieved by leaning forward. He had no history of recent trauma, prior surgeries, or cardiovascular disease. Physical examination revealed cervical lymphadenopathy, persistent tonsillar erythema with swelling, and palpable hepatosplenomegaly. Electrocardiography was unremarkable (Supplemental Figure 1). During attempts to undergo computed tomography (CT), his chest pain was exacerbated in the supine position, and he became severely dyspneic, requiring intubation with mechanical ventilation. CT of the neck revealed thickening of the uvula and tonsils as well as reactive cervical lymph nodes (Figure 1A and 1B). CT of the chest revealed new trivial pericardial effusion with enhancement and thickening of the pericardial sac (Figure 1C and 1D). Serum troponin levels were normal. Additional testing was concerning for viral infection (Table 1). Given his presentation, physical examination findings, and initial diagnostic workup, the diagnosis of viral pericarditis was suspected. He was subsequently admitted to the intensive care unit for further management.

**Table 1:** Summary of pertinent diagnostic workup for potential causes of pericarditis upon admission

	Test name	Result	Interpretation	Reference range
Infectious	Adenovirus PCR	Not detected	Negative	-
	Lyme disease antibodies, IgM	0.67 LIV	Negative	0.00–1.20 LIV
	Coxsackie A9 virus IgG	<1:8	Negative	<1:32
	Coxsackie B antibodies 1–6	<1:10	Negative	<1:10
	Cytomegalovirus PCR	Not detected	Negative	-
	EBV EA antibodies, IgG	28.4 U/mL	Positive	0.0–10.9 U/mL
	EBV NA antibodies, IgG	20.9 U/mL	Positive	<17.9 U/mL
	EBV VCA antibodies, IgG	174.0 U/mL	Positive	0.0–21.9 U/mL
	EBV VCA antibodies, IgM	<10.0	Negative	0.0–43.9 U/mL
	Echovirus PCR	Not detected	Negative	-
	Enterovirus PCR	Not detected	Negative	-
	HIV 1–2 antigens/antibodies	Nonreactive	Negative	-
	Influenza A/B PCR	Not detected	Negative	-
	Metapneumovirus PCR	Not detected	Negative	-
	Non-SARS coronavirus PCR	Not detected	Negative	-
	Pan-cultures	No growth	No growth	-
	Parainfluenza PCR	Not detected	Negative	-
	Rapid plasma reagin	Nonreactive	Negative	-
	Rhinovirus enterovirus PCR	Not detected	Negative	-
	RSV PCR	Not detected	Negative	-
Autoimmune	ANCA IFA	<1:20	Negative	<1:20
	Antinuclear antibodies, IgG	Not detected	Negative	-
	Double-stranded DNA IgG	2 IU	Negative	0–24 IU
	Histone antibodies, IgG	0.4 units	Negative	0.0–0.9 units
	Rheumatoid factor	4 IU/mL	Normal	<15 IU/mL
Miscellaneous	ANC	13.0 thousand/mm^3^	High	1.8–7.7 thousand/mm^3^
	BUN	11 mg/dL	Normal	7–22 mg/dL
	CRP	13.58 mg/dL	High	<1.0 mg/dL
	ESR	5 mm/h	Normal	0–10 mm/h
	Lymphocytes	40.9%	High normal	20%–40%
	Lymphocytes (atypical)	Few	Positive	0
	Neutrophils	45.2%	Normal	40%–60%
	Pro-BNP	99.8 pg/mL	Normal	0.0–124.0 pg/mL
	Troponin T	<0.010 ng/dL	Normal	<0.010 ng/dL
	WBC	21.7 thousand/mm^3^	High	4.8–10.8 thousand/mm^3^

ANC = absolute neutrophil count; ANCA = anti-neutrophil cytoplasmic antibodies; BNP = brain natriuretic peptide; BUN = blood urea nitrogen; CRP = C-reactive protein; EA = antibodies to early (D) antigen; EBV = Epstein-Barr virus; ESR = erythrocyte sedimentation rate; IFA = indirect immunofluorescence; LIV = Lyme index value; NA = nuclear antigen; PCR = polymerase chain reaction; RSV = respiratory syncytial virus; VCA = viral capsid antigen; WBC = white blood cells.

**Figure 2:**
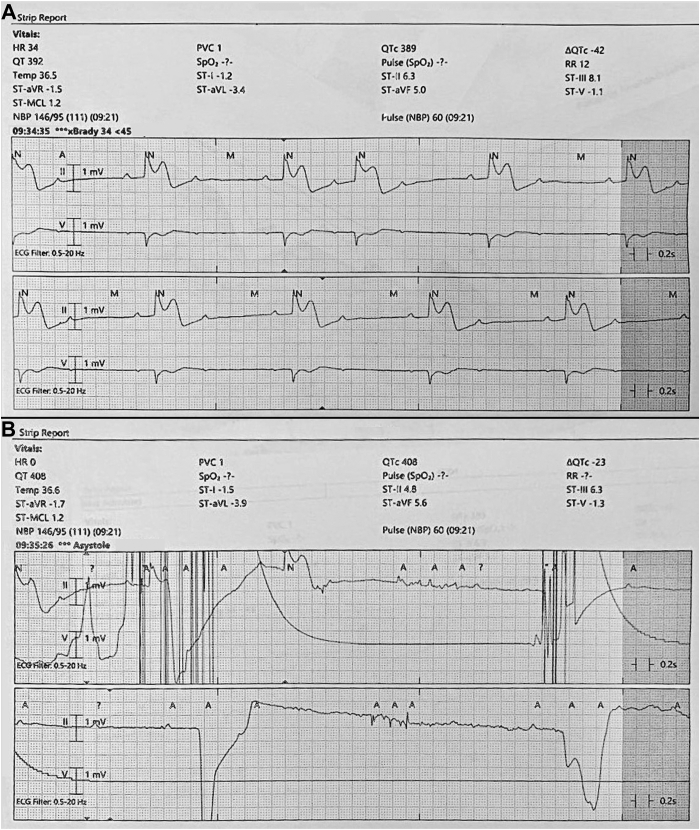
Telemetry strip revealing ST elevations in lead II with Mobitz type II second-degree atrioventricular block (**A**), which rapidly progressed to complete heart block and asystole (**B**).

**Figure 3:**
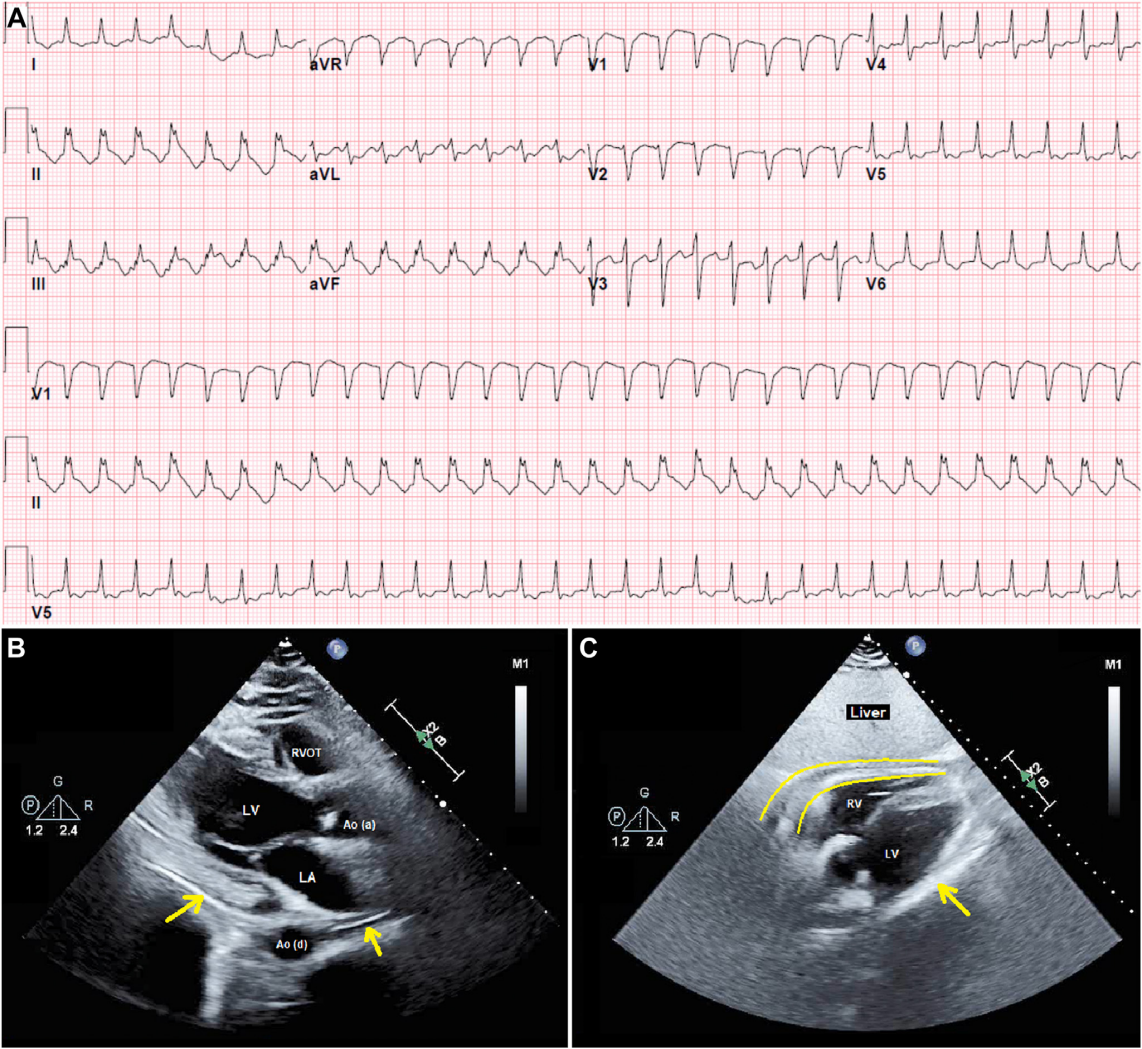
**A:** Electrocardiography after return of spontaneous circulation revealing sinus tachycardia with ST elevations in inferior leads, ST depressions in anterolateral leads, and diffuse T-wave inversions. **B:** Transthoracic echocardiography with a parasternal long-axis view revealing a trivial amount of pericardial effusion. **C:** The subcostal view reveals a hyperechoic thickening of the pericardium consistent with inflammation. Ao(a) = ascending aorta; Ao(d) = descending aorta; LA = left atrium; LV = left ventricle; RV = right ventricle; RVOT = right ventricular outflow tract.

Shortly upon arrival to the intensive care unit, he became bradycardic and decompensated into cardiac arrest. Cardiopulmonary resuscitative measures were successful after 4 minutes. Retrospective review of his telemetry revealed ST elevations with type II second-degree heart block (Figure 2A), which progressed to third-degree (complete) heart block and asystole (Figure 2B). Postresuscitative electrocardiography revealed sinus tachycardia with ST elevations in inferior leads, ST depressions in anterolateral leads, and diffuse T-wave inversions (Figure 3A). However, cardiac catheterization revealed no evidence of obstructive coronary artery disease (Supplemental Figure 2). Intraprocedural echocardiography revealed trivial anterior pericardial effusion with an ejection fraction of 55% (Figure 3B and 3C). Serum troponin levels were yet again normal. The patient was provided with high-dose ibuprofen and a transvenous pacemaker, which remarkably improved his symptoms. Thorough workup for etiologies of his complete heart block revealed antibody serologies consistent with a subacute EBV infection (Table 1). Serum liver enzyme function tests levels were all normal. Owing to the unpredictable nature of his course and the concern for future reactivation of EBV or progression to chronic state of disease, decision was made on day 4 to exchange the transvenous temporary pacemaker with a leadless Micra™ AV permanent pacemaker (Medtronic, Dublin, Ireland), as he would be at risk for having further events. The Micra was selected owing to his normal sinus node function and the expectation of little to no ventricular pacing in him. After a total of 7 days of hospitalization, he was safely discharged home in stable medical condition.

### Follow-up

The patient is now 3 months status post pacemaker placement and has done very well. A final 3-month device report revealed a right ventricular pacing impedance of 640 Ω, a capture threshold of 0.38 V at 0.24 milliseconds, a sensed R-wave amplitude of 14.2 mV, and no further episodes of arrhythmias, thus pointing to the likelihood that his conduction disturbances were transient and reversible.

## Discussion

Pericarditis is a complication associated with many viral infections and is the most common inflammatory heart disease. It is diagnosed in as many as 5% of patients presenting to the emergency department with nonspecific angina. The diagnosis of viral pericarditis in our case was largely clinical and based on the presence of sharp pleuritic chest pain exacerbated by lying supine and on deep inhalation, pericardial rubs on auscultation, and thickening of the pericardium in the setting of a known recent viral infection. Elevated C-reactive protein levels and lymphocytosis, though nonspecific, are also consistent with this diagnosis. It is important for clinicians to recognize and treat this inflammatory disorder early on to prevent progression and life-threatening complications such as constrictive pericarditis, pericardial effusion, and cardiac tamponade.^6^

Several immunologic markers are used to determine the timeline of EBV’s infectious course. EBV viral capsid IgM antibodies (VCA) appear very early in infection and typically disappear within 6–8 weeks as VCA IgG will remain elevated indefinitely. EBV nuclear antigen (EBNA) antibodies are not seen in the acute phase of infection but slowly appear after a couple months of transmission. Anti–early antigen (EA) IgG antibodies also appear in the acute phase of infection but begin to disappear within 6 months. Therefore, elevated serum levels of VCA IgG, EA, and EBNA antibodies with concomitant normal levels of viral capsid IgM antibodies suggest that our patient had either an EBV reactivation in an immunosuppressed state or recent EBV infection that is no longer acute.

EBV, coxsackie, cytomegalovirus, and influenza are among the more commonly known viral etiologies of viral pericarditis. Although the risk of viral pericarditis progressing to severe pericardial effusion, myocarditis, tamponade, arrhythmias, or sudden cardiac death is well recognized, it is very unique to see this case of subacute EBV pericarditis leading to complete heart block, as this is typically a life-threatening complication once there is progression to myocarditis and is associated with a much higher morbidity and mortality. The ST-segment elevations immediately pre and post arrest with concomitant high-degree AV block and nonobstructive heart catheterization findings suggests that either coronary vasospasm was occurring, which led to transient nodal ischemia, or the node itself was experiencing block due to infection-induced inflammatory changes. Irrespective of the definitive sequelae, both possibilities should be considered by clinicians.

Pathologically, AV blocks are highly associated with pericardial and myocardial fibrosis or sclerosis. Thus, in our case, we believe the long-standing pericarditis is culprit to the cardiac sequelae that ultimately led to cardiac arrest. AV block and/or coronary vasospasm would also explain why he became so dyspneic upon lying supine during CT imaging. Complete heart block is one of the rare complications of vasospastic angina and can lead to symptoms like dizziness and shortness of breath. A range of 5%–10% of all inferior myocardial infarctions result in high-grade or complete heart block.^7^^,^^8^ We believe the persistent inflammation led to electrical conduction disturbances and possibly transient vasospasms, which caused our patient’s severe dyspnea on arrival. We were fortunate enough to capture his arrhythmias and heart block on telemetry and definitively treat him with implantation of a permanent pacemaker. Upon placement of the pacemaker, his symptoms completely resolved.

Typically, reversible causes of heart block must be treated or ruled out prior to committing to a permanent pacemaker. In these cases, temporary pacing may be reasonable until the underlying cause is managed and heart block resolves. However, as in our case, symptomatic bradycardia and refractory or irreversible causes of heart block require a permanent, implantable pacemaker.^9^ Such patients should be monitored outpatient as this subacute or refractory infection can progress to nasopharyngeal carcinoma or Burkitt lymphoma. They should be monitored for fever, malaise, hepatosplenomegaly, coagulopathy, cytopenia, and unintentional weight loss.

The limitation of our case is that we were unable to definitively determine if our patient’s cardiac arrest was directly due to complete heart block, an underlying coronary vasospasm, or some combination of both. Perhaps an intraprocedural provocation test for coronary vasospasm during angiography may have offered more insight on this consideration. Additionally, despite normal troponin levels and ejection fraction, cardiac magnetic resonance imaging would have helped definitively rule out subclinical myocarditis, a known etiology of reversible sick sinus with heart block. Nonetheless, it is still important for clinicians to recognize EBV not only as a cause of pericarditis, but also as one that can progress to life-threatening complications such as coronary vasospasm, electrical conduction disturbances, complete heart block, and sudden death. Larger-scale studies are needed to allow clinicians to better understand such life-threatening manifestations of EBV.

## Figures and Tables

**Supplemental Figure 2:**