# Supplementary material for: A case of Epstein-Barr virus–associated pericarditis progressing to complete atrioventricular block and cardiac arrest
Source: HeartRhythm Case Rep. 2023 Jul 23;9(10):709–14. doi: 10.1016/j.hrcr.2023.07.010 (PMC10691948; doi:10.1016/j.hrcr.2023.07.010)
Supplement: Supplementary Figure legends [file mmc1.docx]

**Supplemental Figure 1:** ECG on admission revealing normal sinus rhythm with nonspecific T wave inversions in leads aVL and V1 but no ST changes.

**Supplemental Figure 2:** Angiography of the right (Panel A) and left (Panel B) coronary arteries revealing widely patent vessels with Thrombolysis in Myocardial Infarction (TIMI) Score of 3 and no evidence of any obstructive disease.
